# Enhanced Capacity to Act: Managers’ Perspectives When Participating in a Dialogue-Based Workplace Intervention for Employee Return to Work

**DOI:** 10.1007/s10926-020-09914-x

**Published:** 2020-07-31

**Authors:** Therese Eskilsson, Sofia Norlund, Arja Lehti, Maria Wiklund

**Affiliations:** 1 Department of Community Medicine and Rehabilitation, Section of Physiotherapy, Umeå University, Umeå, Sweden; 2 Department of Public Health and Clinical Medicine, Section of Sustainable Health, Umeå University, Umeå, Sweden; 3 Department of Clinical Sciences, Section of Professional Development, Umeå University, Umeå, Sweden

**Keywords:** Workplace intervention, Return to work, Exhaustion, Burnout, Qualitative research

## Abstract

*Purpose* To explore if and how a dialogue-based workplace intervention with a convergence dialogue meeting can support a return to work process from the managers’ perspective. *Methods* Individual interviews were conducted with 16 managers (10 women and 6 men) who had an employee on sick leave because of stress-induced exhaustion disorder. The manager and employee participated in a dialogue-based workplace intervention with a convergence dialogue meeting that was guided by a healthcare rehabilitation coordinator. The intervention aimed to facilitate dialogue and find concrete solutions to enable return to work. The interviews were analyzed by the Grounded Theory method. *Results* A theoretical model was developed with the core category e*nhancing managerial capacity to act in a complex return to work process,* where the managers strengthened their agential capacity in three levels (categories). These levels were *building competence, making adjustments, and* s*haring responsibility* with the employee. The managers also learned to navigate in multiple systems and by balancing demands, control and support for the employee and themselves. An added value was that the managers began to take preventive measures with other employees. When sick leave was caused only by personal or social issues (not work), workplace actions or interventions were difficult to find. *Conclusions* From the managers’ perspective, dialogue-based workplace interventions with a convergence dialogue meeting and support from a rehabilitation coordinator can strengthen managerial competence and capacity to act in a complex return to work process.

## Introduction

Work-related stress and burnout are common and increasing in Europe, with serious consequences for both the individual and society at large [1]. In Europe, 25% of employees experience work-related stress most of their working time, and a similar proportion indicate that work negatively affects employee health [2], which may increase the risk of sick leave [1]. A large proportion (80%) of European managers are concerned about the problem of stress among employees [2], and the financial burden of sick leave due to work-related stress is significant for society and the workplace [3].

In Sweden, 28% of employees reported ill health caused by work during a 12-month period, and nearly one third of these were absent from work. Excessive workload was the most common cause, with symptoms of fatigue, pain, sleep disturbances, cognitive impairments, anxiety, depression and exhaustion [4]. In Sweden, adjustment disorder and reaction to severe stress, including stress-induced exhaustion disorder (SED) [5] are the most common reasons for sick leave since 2014 [6]. These diagnoses are also associated with long-term sick leave [6]. SED is classified as an illness in the Swedish version of ICD-10 and seems to be the most valid clinical equivalent of burnout [5, 7]. The primary symptoms of SED are markedly reduced mental energy, lack of endurance and increased recovery time after mental effort [5]. Somatic [8] and mental symptoms [9] are common, as well as cognitive impairments in areas of memory, attention and executive functioning [7], and cause considerable impairment in social and working life.

Return to work (RTW) for persons on sick leave because of SED is often a problematic and prolonged process [6]. This may be because the focus is primarily on the individual [10]. In contrast, interventions involving the workplace seem to positively affect sick leave among employees with mental health problems, although some findings are somewhat inconsistent [11–14]. For SED, workplace interventions are key to successful RTW [5, 15]. In order to involve the workplace, a workplace-oriented intervention has been developed in Sweden for persons on sick leave due to SED [16]. The intervention is a 3-step interview model including a convergence dialogue meeting (CDM). A health care provider coordinates the CDM to initiate dialogue between the employee and the manager in order to find suitable solutions for RTW [16]. The 3-step interview model shows positive effects on RTW for persons with SED [16, 17] and over time among younger participants [18]. In a recent study, the CDM was developed further by including a health promotion approach that focuses on the work environment when discussing work tasks in relation to work ability. In that study, employees on sick leave with SED experienced this dialogue-based workplace intervention as health promoting, as it enhanced communication and collaboration with the manager and other involved stakeholders, and supported RTW [19].

Work-related stressors such as quantitative and emotional demands are common among persons with SED [20]. This is why it is important to facilitate dialogue and actions at the workplace. Relationships between psychosocial risk factors at work (such as high demands, low job control, high work load, low reward and job insecurity) increase the risk for developing exhaustion, while job support and work fairness are protective [21]. Regardless of work support, high psychological demands are associated with exhaustion [22]. This shows the importance of focusing on adjustment of job demands to prevent symptoms of exhaustion in the RTW process [22]. In Sweden, employers are responsible for promoting a good work environment and preventing risks of ill health that are due to organizational and social work conditions [23]. Employers are also obligated to plan for RTW for employees who have been absent for 30 days due to incapacity to work and who can be assumed will be absent from work for more than 60 days [24]. Despite this responsibility, managers state that mental health problems among employees are complex and they need knowledge and strategies for how to provide support in these cases. They also want support and collaboration from other actors in the process [25].

Several actors from the work, health, and insurance systems are involved in the RTW process [26]. However, there is a service and knowledge gap in the RTW process of persons with mental health problems [27], as well as between the different systems [28], that can make the RTW more difficult. Strategies that facilitate RTW are clarity in the actors' roles and actions, and coordination between the systems [26]. The role of RTW coordinators is important in facilitating communication between the various actors and systems [26, 28, 29]. To promote communication and support between the workplace and people on sick leave, healthcare in Sweden has introduced rehabilitation coordinators in both primary and specialty healthcare [30]. Research that describes the effects of the rehabilitation coordinator’s role in RTW is recent and limited [29, 31]. Therefore, it is important to use empirical research to investigate how managers perceive participation in workplace interventions provided by healthcare for persons with SED. This study explores if and how a dialogue-based workplace intervention with a convergence dialogue meeting can support the RTW process from the manager’s perspective. Based on the results, a theoretical model that contextualizes managerial involvement in the RTW process will be developed.

## Methods

### Study Design

This study uses a qualitative research design. Individual interviews with managers were performed and analyzed using a social constructionist grounded theory approach according to Charmaz [32, 33]. This approach was chosen to gain contextualized insights into the managers’ perspectives when participating in the intervention. Grounded in data, it guides the development of a theoretical model, or ‘interpretative theory’, that conceptualizes the studied phenomenon to understand it in abstract terms [32]. Grounded theory allows a focus on experiences, actions and processes, in context and over time. To note, a social constructionist approach to research acknowledges participants’ and researchers’ subjectivity and background.

### Study Setting and Participants

Participants are first-line managers responsible for rehabilitation at the workplace. The managers were recruited because they had an employee diagnosed with SED who was on at least 50% sick leave and had participated in a 24-week multimodal rehabilitation (MMR) at the Stress Rehabilitation Clinic at the University Hospital in Umeå, Sweden. The MMR program included group-based cognitive behavioral therapy to support behavioral changes for persons with SED, as previously described [34], and a dialogue-based workplace intervention. The MMR program has a multidisciplinary team led by a physician, psychologist/psychotherapist, physiotherapist and rehabilitation coordinator. After completion of the MMR program, 22 managers of the MMR program participants were asked to participate in individual research interviews. Purposive sampling was used to provide variation in age, gender, years of managerial experience, and work sector (governmental, county council, municipal or private). Six managers declined to participate and this resulted in 16 remaining managers (10 women and 6 men) who agreed and were interviewed. The managers were 27–62 years of age, and had less than a year to 30 years of managerial experience. Eight participants worked in the private sector, three in the county council sector, three in the municipal sector, and two in the governmental sector (Table 1).

**Table 1:** Characteristics of managers interviewed

	*n* = 16
Gender, *n*	
Women	10
Men	6
Age, mean, years	45.7
Work sector, *n*	
Governmental	2
County council	3
Municipal	3
Private	8
Manger experience, *n*	
1–2 years	8
3–5 years	3
10–15 years	3
30 years	1

### Dialogue-Based Workplace Intervention with a Convergence Dialogue Meeting

The aim of the workplace intervention was to facilitate dialogue between the employee and the manager to support the employee’s RTW. The focus of the intervention was to strive for converging perspectives and goals between the manager and the employee [16]. In a 3-step interview model, a rehabilitation coordinator guided the dialogue, and included one or more follow-ups as needed. The rehabilitation coordinator performed structured interviews, first with the employee and then with the manager. Both responded to the same questions about expectations and concerns for rehabilitation, perceived main cause for the sick leave, possible work task adjustments that could facilitate RTW, and motivation and confidence in RTW. The manager also answered questions about access to occupational health services and whether actions were planned. In the third step, a CDM was held where the rehabilitation coordinator, employee and manager discussed solutions to enable RTW. The rehabilitation coordinator had specific knowledge of SED and was able to give concrete advice on adjustments for this disease. The CDM built upon a health promotion approach, and focused on the work environment (physical, organizational and social factors) when discussing work tasks in relation to the employee’s work ability. Based on this, individual work task adjustments could be identified and documented in a written plan that divided responsibility between the employee and the manager. The manager was thereafter responsible for initiating continuous follow-ups and revision of the plan if goals or actions needed adjustment. Involved actors, including the Social Insurance officer, received a copy of the written plan. The rehabilitation coordinator also had the opportunity to get support from the MMR multidisciplinary team. The intervention and health promotion approach have been described in detail previously [19]. In that study, the manager is described as a supervisor.

### Data Collection

The research interviews were conducted after the intervention and MMR program had ended. Two psychologists, experienced in interview techniques and analysis of personal accounts, performed the interviews. Neither of them was involved in the employees’ rehabilitation. Three of the interviews were performed by phone, one by video-link, one at the manager’s workplace, and the rest at the Stress Rehabilitation Clinic. The interviews followed a semi-structured interview guide where the managers were asked to reflect on experiences and outcomes of the dialogue-based workplace interventions and their role in this, including perceived barriers and facilitators to the RTW process. The interviews lasted between 35 and 75 min, were audio-recorded, and transcribed verbatim.

All participants received written and verbal information about the study, and gave their written informed consent. The Regional Ethical Review Board in Umeå, Sweden (Approval No. 2015/49-31Ö) approved the study.

### Data Analysis

The analytical procedure started in parallel to the data collection, consistent with the chosen grounded theory approach [32, 33]. Following Charmaz [32, 33], we focused on ‘experiences’, ‘actions’ and ‘processes’ to capture participant perspectives from taking part in the intervention. Throughout the procedure, analytical written notes (memos), discussions, and triangulation between researchers with different expertise and perspectives were central [33]. After each interview, the interviewers made memos about the content and analytical thoughts, and discussed these with the first author. In the following process of constant comparison [32, 33], we continuously compared memos, codes, categories, and each interview with our interpretation as a whole.

**Fig. 1:**
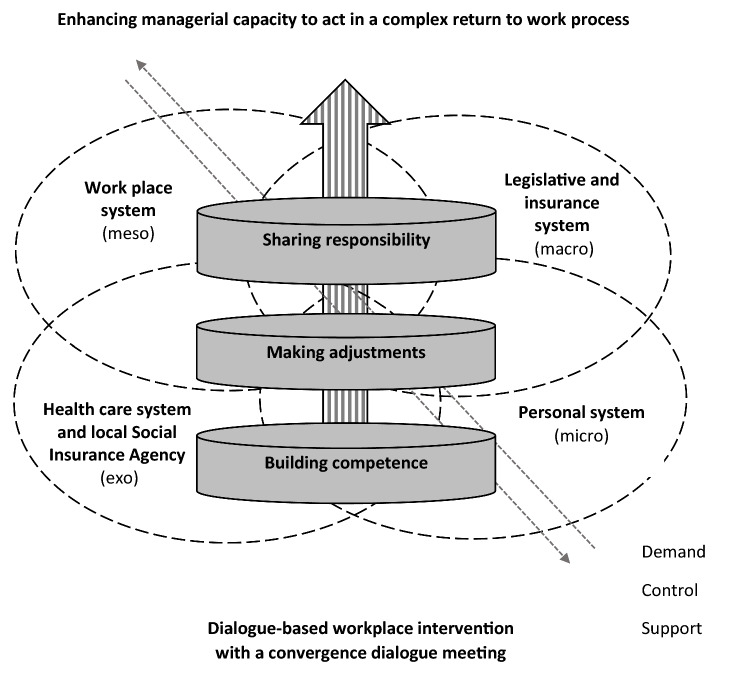
Theoretical model illustrating managers’ enhanced capacity to act in a complex return to work process over time and in context

Initially, as part of the *open coding* [32, 33], each of the four authors independently read and coded the same interview (line-by-line and in-vivo), and compared emerging analytical thoughts. The open coding stayed close to the data with a limited degree of abstraction. When creating codes capturing actions or processes, words ending in ‘ing’ were used (e.g. making, building, learning, balancing). Then the two first authors continued by independently coding and discussing two additional interviews. Thereafter, they read and coded seven (TE) and six (SN) interviews before they met and started the *selective coding*. This meant that they clustered codes with similar content into preliminary categories on a more abstract level [32, 33]. Thus, the empirical data was synthesized in a new way; whereby key-actions and processes were identified and conceptualized. From the open and focused coding, three categories with interrelated sub-categories were developed that represented participant competencies and agential capacities (actions, processes). In a process of *axial coding* [32], the categories were compared on how they related to each other in a process over time (the intervention). In doing so, the three categories served as ‘building blocks’ for conceptualizing a key process, the core category. During the following *theoretical development*, the key process with its building blocks (categories) was *contextualized* by relating it to additional ‘actors/actions’, ‘processes’ and ‘systems’ identified in the empirical material (e.g., micro–meso–exo–macro; demand–control–support). Our theoretical model is presented as a figure, illustrating the key process in context and over time (Fig. 1). The final step of creating ‘interpretative theory’ [32], the integration with existing concepts and theories, is presented in the discussion.

The research team consisted of one physiotherapist (TE) and one physician (AL), with specific expertise and research in work-related rehabilitation of SED. The physician and a second physiotherapist (MW) had expertise and research in the field of mental health and prolonged pain, equal care, medical sociology and gender studies. One sociologist (SN) had specific competence in social theory, and psychosocial and work-related perspectives on burnout. None of the authors were involved in the intervention or had met the participants’ employees as patients at the clinic.

## Results

The data analysis resulted in a core category, enhancing managerial capacity to act in a complex RTW process, that was comprised of three categories: building competence, making adjustments, and sharing responsibility (Table 2). The three categories, with interrelated sub-categories, should not be viewed as separate from each other since they are intertwined and build upon each other as three ‘building blocks’ forming the key process (core category).

**Table 2:** The analytical process that resulted in a core category, three categories and seven sub-categories

Core category	Enhancing managerial capacity to act in a complex return to work process		
Categories	Building competence	Making adjustments	Sharing responsibility
Sub-categories	Recognizing and dealing with signs of stress Refining communication Learning about the multifaceted rehabilitation process	Balancing workload Creating clarity by a written plan	Enabling mutual responsibility Promoting trust and an honest dialogue

### Enhancing Managerial Capacity to Act in a Complex Return to Work Process

Our theoretical model (Fig. 1) captures how the managers developed competence and agential capacity by participating in the dialogue-based workplace intervention with a convergence dialogue meeting with their employee, guided by the rehabilitation coordinator. This key process, the core category, is formulated as *enhanced managerial capacity to act in a complex RTW process*. Enhancing agency by *building competence*, *making adjustments* and *sharing responsibilities* was important from the managers' perspective. Managers felt that the meetings, both the one-on-one with the rehabilitation coordinator and the one with the employee, gave them a solid basis for an open and candid relationship with the employee in the challenging RTW process. In this way, the managers enhanced their capacity to act with mutual respect in a realistic manner.

Our theoretical model illustrates the *context* and *complexity* of the RTW process, and places the managerial capacity to act in a multifaceted context of ‘rules and resources’ at different arenas and levels in society (micro, meso, exo, macro). During the intervention, managers learned to navigate and balance demands, control and support for the sake of their employees and themselves. In doing so, they saw the advantage of bringing actors and resources together, and how that bridged different systems: the personal system (micro), the workplace system (meso), the health care system and local Social Insurance Agency (exo), and the legislative and national insurance system (macro). Their bridging of actors and systems became integral to their competence, and enhanced agential capacity, together with their balancing of demands, control and support (Fig. 1).

Factors such as the manager’s personality and individual competence (micro–meso), as well as aspects such as leadership style, and organizational structure (meso) influenced the degree to which the manager felt the need for support in the employee’s RTW process. Also, aspects linked to the individual employee’s health and social situation (micro–meso), together with ‘rules and resources’ linked to health services, the work environment, or the national social insurance system (meso-exo-macro), were seen as influencing the complexity of the RTW process. Before entering the intervention, some managers had contacted actors such as human resources or occupational health services for support in the employee’s rehabilitation. Others already had a close and continuous dialogue with their employee, making their perceived need for support from the dialogue-based workplace intervention less important. Managers new to managerial positions expressed more need of the structure provided by the intervention. However, even managers with many years of experience in rehabilitation and RTW processes stated that they gained knowledge from engaging in the intervention since it gave them an opportunity to refine their competences and strategies.

### Building Competence

Building competence was the first building block and a central outcome generated by the managers’ active engagement in the intervention and guided by the rehabilitation coordinator. Building competence involves gaining knowledge and capacities that are crucial and specific for rehabilitation of SED, such as recognizing and dealing with signs of stress, learning about the rehabilitation process, and refining communication with the employee on sick leave, other employees at the workplace, and external actors (bridging micro–meso–exo–systems). Managers thought it was crucial that the rehabilitation coordinator be an expert in different areas such as SED and rehabilitation medicine, because this facilitated the process. The managers thought it was positive to have somebody to talk to and somebody who listened to their concerns. Previously, they had not been able to explain their particular situation and perspective.

The conversation with the rehabilitation coordinator before the three party dialogue [CDM] is crucial for understanding what kind of person I am and what requirements I have … and then you are really grateful if the rehabilitation coordinator tells you what we can or cannot achieve, or says what you have to resolve. (Manager 10)

*Recognizing and dealing with signs of stress* captures how the managers learned to pay attention to early signs of employee stress and overload. If the manager had personally experienced SED, they found it easier to see the warning signs. They perceived it as more difficult to detect signals of stress and mental illness than of physical problems, and that it was especially difficult when the employee performed well at work and displayed a happy countenance.

It’s easy to look at someone who has a leg fracture, and ‘well, you can't go up the stairs, okay, we have to take the elevator’. But a person who is burnt out or depressed, more mentally unstable, can look very happy and normal, and then you don’t understand. (Manager 5)

Through the intervention, managers also learned that other types of questions are needed to capture potentially ‘unhealthy’ workloads and subtle signs of stress (compared to physical work environment). This was particularly seen among high-performing employees who did not signal ill health, and in this situation it became important for the manager and employee to discuss and reach consensus on a reasonable workload.

From the outside, he is very skilled….He manages his projects well, is not stressed, does not talk fast, does not rush, looks good, exercise regularly, and does not exaggerate much. But now with the facts in hand, and now with even more experience [after the intervention], I notice that he has a huge need for control, enormous loyalty, loves what he does. Therefore, he can never turn off because it’s so much fun. That’s an experience I’ll bring with me, and ask other questions. For the questions I [previously] asked about workload… well… his own frame of reference on what could be done in one day was completely distorted. (Manager 15)

*Refining communication* was facilitated by manager engagement in the convergence dialogues, and contributed to their building competence and agential capacity. Examples of insights the managers gained were how to pose questions and talk about feelings and psychological views without it being “so serious*”* (Manager 2). Gaining knowledge and dealing with the employee’s stress also involved a plan for how to communicate and inform co-workers about the employee's current situation. This was a way to prepare for the employee to RTW, and was expressed as *“raking the way”* (Manager 1). The managers learned strategies to involve the employee in the workplace despite their absence. For example, the employee could be invited to workplace meetings or be updated. Managers also learned the importance of dialogue, in order to balance the employee’s need for recognition during the process of reintegration into the workplace.

The [workplace] dialogue is a balancing act, not giving out too much, but constantly giving support. ‘What do you want to lift up? What do you dare? What do you want to say?’ Sometimes, as a manager, you know much, much more than you can say. You can’t, and then you run the risk of putting the employee off balance. Yes, I think it is healing to feel welcome. (Manager 1)

*Learning about the multifaceted rehabilitation process* was enhanced by becoming aware of how to act as a manager in relation to wider rules and regulation in the Swedish welfare system, as well as to best practice and evidence-based guidelines of rehabilitation tied to SED. With support from the rehabilitation coordinator, and based on the specific workplace conditions, the managers appreciated how they were gradually guided into principles of the rehabilitation process:

I wasn't prepared for that, but I learned from the process, the more I got to talk and hear. (Manager 14)

Managers also increased their knowledge of the social insurance system and how to navigate the rules and resources (macro-system). On their own, they found it difficult to understand and be updated on the rules about medical coverage and sick leave. Therefore, this guidance was seen as an important prerequisite for taking action in a well-informed manner. However, if the manager thought that the reason for sick leave was primarily based in the employee’s private life, difficulties could arise.

I thought everything [in the intervention] was focused on returning to work. I think the first priority for the employee is the family. Because that’s still the most important thing in your life. It’s not the job. If you can’t manage the home situation, (such as) taking care of your children, how do you manage to do a job? Step two is to get out into working life. (Manager 9)

The above quote illustrates how the managers were aware of the multifaceted etiology of SED, including the interplay between personal conditions and working life (micro–meso-systems). Overall, the managers thought they gained support from the intervention in managing this, although a few questioned the company’s responsibility if the cause of SED was understood to be primarily social.

### Making Adjustments

*Making adjustments* was the second building block that generated managerial agential capacity in the multifaceted RTW process. Managers learned that they needed competence to make tailored adjustments dependent on the individual employee’s health and social situation and the specific work place conditions (bridging micro–meso-systems). Such adjustments also involved exo-level actors such as the local Social Insurance Agency and local health services. Competence meant being able to see the individual employee in context and “go from there”. Making adjustments therefore built upon increased managerial competence regarding stress, rehabilitation and communication, which also incorporated the importance of sustainability in balancing demands, support and control for the sake of the manager and employee. The adjustments were discussed primarily in the CDM, where the rehabilitation coordinator played an important role. That the rehabilitation coordinator had heard both sides and could guide the manager and the employee in the conversation was perceived as important. Managers felt that they were guided with openness, within clear boundaries, and had the opportunity to influence and control the adjustments in relation to their own work organization.

Yes, I thought it was appealing, concrete and clear, but still with an openness so I could be involved and influence [the adjustments]. So very professional that I immediately felt a sense of security and felt that this was good. It was solid advice, but we still had to come up with it ourselves, to place it in our context. (Manager 2)

*Balancing workload* describes how the manager learned tools to adapt work tasks to the employee’s abilities. This gave both the manager and the employee a sense of security. Previously, some managers had given their employees *“free rein”* in the belief that doing so would reduce stress. Instead, the employees experienced more stress from unclear boundaries and frameworks, and this was counterproductive in the overall RTW process.

Through the dialogue-based workplace intervention, managers perceived that they learned the extent to which they could put demands on their employees and dare to “push just the right amount”. Another common adjustment they started to use was to clarify the work content. This was perceived as automatically reduce the employee’s workload by providing them with control. Cognitive impairments were a common challenge for managers when employees had SED. Flexible office space was perceived as extra challenging for employees with cognitive impairment, and managers learned specific adjustments such as access to a separate room for performing tasks requiring concentration.

‘X’ sits in a room and updates written documents. Things that no one has done before. And it’s perfect because it requires accuracy and she can’t handle noise at all. (Manager 7)

*Creating clarity by a written plan* was found to be crucial and something that managers learned to do. They highlighted the importance of being able to continuously adjust the plan (e.g., work tasks, schedules) depending on changes in the employee’s situation and health. Both the employees and managers used the written plan as a structured and flexible tool.

I thought it was structured and there was a plan all the time. We adjusted it during the process, depending on how the load was …and I thought it was very good. (Manager 14)

Carrying out regular follow-up of the plan was one of the most important strategies that managers thought they learned. Regular follow-ups were particularly important if the plan was not followed. However, one of the managers (Manager 9) thought that the RTW process should end when goals were not achieved after repeated attempts.

As an added value, some of the managers started to be pro-active and transferred their new strategies into situations with other employees who were close to, or back from, sick leave. A manager with long experience said:

What I bring [from the intervention]….is to make written agreements with specific things that we have decided, and to follow up. (Manager 1)

Creating clarity, making adjustments, and taking multiple complexities into account thus became an ‘added value’ that was incorporated in the daily managerial practices and ‘tool box’.

### Sharing Responsibility

*Sharing responsibility* was identified as the third building block in the process, where managers strengthened their competence and capacity to act. In planning rehabilitation and in the actual rehabilitation, managers felt it was important to *enable mutual responsibility*. This was facilitated by the dialogue-based intervention with open and structured communication. Managers thought it was vital to understand what responsibilities each person had in the process: manager, employee, and other external actors (bridging meso-exo-systems). The managers felt that the increased participation of all parties gave joy to the employee, which meant that self-confidence increased throughout the RTW process and the managers could take more of a coaching role.

Managers wanted to define the different actors’ responsibilities, particularly as they perceived that other actors had a big impact on the outcome of the RTW process and therefore should be represented. Managers thought the plan would ideally involve the local Social Insurance Agency and medical professionals, and a few also mentioned that the partner of the employee is important to involve in rehabilitation planning. They thus acknowledged the value of coordinating with involving actors from different systems (Fig. 1).

… To find this golden middle ground is what you should achieve in the dialogue conversation. To have a shared timeline ahead. What will happen… (Manager 10)

*Promoting trust and an honest dialogue* was seen as important. Overall, the openness and transparency that the dialogue led to was appreciated. The managers stated that they had improved relationships with the employees in the sense that they were more open and knew what the other felt and thought. Often the managers realized that they and the employee had a similar perception of the situation and this was something that they did not always think at the beginning. In one case, however, the manager felt that the rehabilitation coordinator became a competitor in the dialogue with the employee, because the employee seemed more comfortable communicating through the rehabilitation coordinator instead of directly with the manager.

Managers stated that when a manager and employee build a trusting relationship, they focus more on the rehabilitation process, and less on how to perceive and interpret the other person's intentions. Another part of the honest and respectful approach was to be frank, realistic, and specific. This meant that the plan was made on realistic grounds and focused on the present work situation at the particular workplace.

I think you should be straightforward and honest, but also understanding…. I think it can help the employee to feel safe and maybe dare to be even more open. Not having to be afraid, getting support. (Manager 11)

Overall, the managers’ experiences of participating in the intervention capture a RTW process—although complex and demanding—characterized by mutual respect between managers and employees that forms a solid common ground for both managerial and employee capacity to act.

## Discussion

This study explores managers’ perspectives of if and how a dialogue-based workplace intervention with a convergence dialogue meeting provided by healthcare, can support the RTW process of employees on sick leave because of SED. Our main results, captured by the theoretical model (Fig. 1), demonstrate that the dialogue-based workplace intervention supported by a rehabilitation coordinator enhances manager capacity to act in the complex and multifaceted RTW process. It provides a solid basis for an open and honest dialogue between the manager and employee. Managers strengthened their agential capacities by building competence, making adjustments, and sharing responsibility with employees.

Our theoretical model, can be primarily understood to illustrate a process of enhanced managerial ‘agency within structures’ [35, 36], referring to the dialectic interplay between individual actions and structuring conditions during the RTW process. According to Zanin and Piercy [37], in the context of mental health services, *structures* can be seen as the rules and resources that ‘enable and constrain decision, choice, action, and thought’ (p. 185). They explain *agency* as the individuals ability to ‘take action’, and this is in line with the definition of agency as the ‘capacity to act’ [38] or more specifically ‘the sociocultural mediated capacity to act’ [39]. Actions are understood to be goal-directed and intentional, connected to reflexivity and rationality. Shapiro [40] concludes that in an agency relationship ‘one party acts on behalf of another’ (p. 263). In our study this can be translated to the managers’ agency on behalf of or together with the employee. The concept of ‘managerial agency’ has previously been used in management and organization studies [41, 42], although in this study (without knowing this) we first developed it inductively as ‘managerial capacity to act’, and then during the stage of theorizing and contextualizing, connected it to the concept of ‘agency within structures’ [35] and Giddens’ ‘structuration theory’ [36].

Our results demonstrate how individual (managerial) agential capacities were mobilized by an intervention guided by the rehabilitation coordinator within (and despite of) a complex context. As outlined by Bronfenbrenner [43] ‘context’ can be differentiated into micro-, meso-, exo- and macrosystems as illustrated in our theoretical model. The systems were thus multiple and complex, comprising both ‘rules and resources’ that the managers in our study navigated and strove to bridge. In Loisel’s conceptual model of RTW [44], these systems can be referred to the ‘personal system/personal coping’ (e.g., social relationships, affective, cognitive, physical), the ‘workplace system’ (e.g., work relatedness, organization, job position), the ‘healthcare system’ (e.g., variety of care management or multidisciplinary teams), and the ‘legislative and insurance system’ (e.g., society’s safety net, regulations and jurisdictions). In our study, the local Social Insurance Agency proved to be a central exosystemic actor, operationalizing ‘regulations and jurisdictions’ set at the macro level. Although Loisel’s [44] conceptual model and systems are developed in relation to RTW and secondary prevention for workers with disability from musculoskeletal pain, we find them useful to apply in relation to manager engagement in employee RTW because of SED. Schultz et al. [45] define Loisel’s conceptual model as ‘a comprehensive ecological/case management model of RTW’ because ‘actions and attitudes of key stakeholders in the occupational disablement process, together with interactions among stakeholders, are critical in conceptualizing RTW’. Schultz et al. [45] conclude that the workplace, the healthcare system, and the compensation system are the most important stakeholders for employees’ RTW. This is congruent with our theoretical model, reflecting central stakeholders/key actors involved in the RTW process, including the managers themselves. To come forward in the RTW process, the managers engaged in ‘bridging’ with the central stakeholders, who could be both demanding and supportive.

In agreement with Zanin and Piercy [37], we see the exploration of agency-structure dualities through the lens of ‘structuration theory’ [36] as a way to understand *complexity*. In our study, the complexity concerns the RTW process, its actors and context. Tied to managerial agency, the context can be both enabling and constraining. We thus understand managers’ capacity to act as context-dependent, without foreseeing the managers’ individual potentials and responsibilities to take active part in the RTW process. By contextualizing the RTW process, it becomes clear how managers need to navigate multiple and sometimes discrepant demands and interests. Building managerial competences and capacities to handle this may require specific support, as provided by the rehabilitation coordinator in the present intervention. Both managers and employees benefited from taking part in the intervention [19]. This can be compared with the well-established demand-control-support model [46] that focuses on balanced working conditions as crucial for preventing burnout [21].

In the first category of ‘building competence’, the managers described acquiring knowledge in how to detect early signs of stress, especially among high-performing employees and those who show a happy facade. Similar results were found in a meta-synthesis of qualitative research on RTW among employees with mental disorders [28]. That study highlighted how perfectionism, high sense of responsibility, and difficulty in setting limits in demanding work situations can be obstacles in RTW [28]. Since the employee may have trouble seeing their own needs, it is especially important that the manager knows how to detect early signs of stress [26] and to provide timely support. Our results indicate that managers learned how to do this. In our study, captured by ‘refining communication’, managers felt that it was difficult to talk about emotions and psychological factors. This has been reported in other studies, where a manager’s contact with the employee was easier if the employee suffered from musculoskeletal issues rather than mental or stress-related disorders [47, 48]. Important factors that can increase managers’ ability to talk about mental illness are management training on common mental disorders [26, 49], and organizational policies and preventive measures [49]. At an organizational level (mesosystem), education is a strategy to overcome stigma related to mental disorders [50]. This shows the importance of the manager being able to ask about mental illness. From the manager’s perspective, this dialogue-based workplace intervention supported such conversations and contributed to their agential capacity.

As seen in the second building block in the key process of enhancing managerial agency, making work adjustments are important in an RTW processes for which the employer is responsible [23]. In contrast to the present intervention, it has been found that adjustments are rarely based on employee needs or functional limitations [51]. Employers have reported uncertainty regarding work accommodations for employees with mental health problems [25]. Adjusting working conditions during SED is important and this can require specific competence because the employee struggles with long-term recovery. In a recent 7-year follow-up of persons with SED, only 16% reported that they were fully recovered. Common residual symptoms were reduced stress tolerance (73%), extreme fatigue (46%), sleep disturbances (36%), and problems with memory (42%) and concentration (36%) [52]. It is known that, to enable RTW after burnout or SED, it is crucial to lower demands by reducing work pace, workload and conflicting demands at work [22]. It is thus not enough to improve resources such as support and control [22].

In our study, managers strengthened their competence and capacity to make adjustments by participating in the intervention, especially participating in the CDM. The adjustments were concrete, and managers became more confident in setting requirements. Importantly, the adjustments were also made in trustful dialogue with the employees. The guidance by the rehabilitation coordinator, with the specific knowledge of SED on how adjustments could be implemented may have been a success in this intervention. The rehabilitation coordinator also has a close collaboration with both the Social Insurance officer and the multidisciplinary team in the MMR program. Work adjustments are crucial in the RTW process and should involve all stakeholders with a clear description of each actors' role and action [26], which the dialogue-based workplace intervention supported e.g. by the written plan that was perceived as creating clarity. Our results emphasizes the ‘bridging’ of multiple systems (micro–meso–exo–macro) as an important part in the managers’ capacity to act, supported by the intervention and the rehabilitation coordinator.

In our study, the managers thus appreciated the support from the rehabilitation coordinator and the opportunity to express their views. However, it is important for the rehabilitation coordinator to be attentive so as not to become “caught in the middle” between the manager and employee. The rehabilitation coordinator has an important role in ensuring communication, a common understanding between all actors [26, 28], and meaningful cooperation with other involved rehabilitation professionals [53].

As shown in the managerial key process of enhancing agency, honesty and trust during the dialogue is explicitly important for a respectful and mutual RTW process, and essential for achieving shared responsibility. This responsibility was clarified in the written plan and included regular follow-ups. Regular meetings and follow-up of goal attainment are important in a RTW process [54, 55] and associated with a faster RTW for mental health problems [56]. From the managers’ perspective, a challenge in the intervention was when goals and activities could not be achieved despite several attempts. Managers have previously described their uncertainty about how far their responsibility extends for rehabilitation [57], and in this study, participants pointed to this in relation to those cases where employees’s SED was linked to a strained social situation (outside work). Managers are concerned that their own and co-workers’ productivity will be affected when time for support and adjustments is made for employees with mental health problems [25]. A close collaboration between all stakeholders can make the RTW more efficient and sustainable [26].

Despite the many advantages of the intervention, there are also parts that could be further developed. If the sick leave was not work-related, the intervention could include other actors. Because the employee’s whole life is affected in a RTW process, it is important to include the family [54, 55]. In Loisel’s conceptual model [44], this is termed the ‘personal system/personal coping’ and includes social relationships, as well as affective, cognitive and physical aspects. The intervention itself is not an obstacle to involving more actors, and the managers suggested this themselves. Close relatives or the social service can participate in the CDM if needed. This points to the further need of bridging systems as part of the RTW process, as has been integrated into our theoretical model and pointed out by others such as Loisel et al. [44] and Schultz et al. [45]. Moreover, bridging systems and resources are crucial when long-term adjustments such as lowered job demands are required for sustainable RTW and working life. In terms of the agency–structure interplay, our results thus raise central questions about models for shared (social, economic) responsibility—and how far managerial agency and ‘corporate social responsibility’ can extend in making adjustments. However, in a welfare state like Sweden, with a national economic systems of mixed economy (based on both private enterprise and central planning), there is potential to further develop models for ‘bridging’ and ‘sharing’ responsibilities, rules and resources at multiple levels.

Preventing SED is in demand [5, 52]. What was pleasing in this study was that managers developed new strategies on how they could enact preventive strategies through participation in the intervention. For example, they increased their communication and implementation of written plans with concrete actions among co-workers who exhibited some form of ill health. Further research is needed to investigate whether this dialogue-based workplace intervention can prevent the onset of SED.

## Strengths and Limitations

This study has several strengths. To our knowledge, this is the first study that explores, from a manager’s perspective, if and how a dialogue-based workplace intervention with a convergence dialogue meeting provided by a healthcare rehabilitation coordinator can support a RTW process. The managers in this study represent a variety of ages, gender, work sectors, and years of managerial experience. Therefore, we think the results could be transferred to similar settings. The theoretical model, generated by our grounded theory approach, may facilitate further explorations and conceptualizing in other contexts. Another strength is that the psychologists who conducted the interviews were experienced in interview techniques, but not involved in the employees’ rehabilitation. In addition, different disciplines and research fields were represented during the analyses and interpretations and this provided triangulation between researchers’ perspectives [58].

We acknowledge the small sample size and that only the perspectives of managers who participated and who potentially were positive about the intervention were captured. The results also highlight difficulties with the intervention and point to future improvements.

## Conclusions

This study demonstrates that managers enhance their capacity to act in the complex RTW process when they participate in a dialogue-based workplace intervention with a convergence dialogue meeting. From a managerial perspective, building competence, making adjustments and sharing responsibility proved to be important agential capacities when navigating complexity and bridging multiple systems together (micro, meso, exo, macro), towards employees’ RTW. With support from a rehabilitation coordinator, managers increase their knowledge of how to detect early signs of stress and to make concrete work adjustments that are tailored to the employee’s ability, which includes a clear plan with regular follow-ups.

Based on the results, we suggested that managers should be able to request a dialogue-based workplace intervention from healthcare providers or occupational health service when they have an employee who is sick or is at risk of being sick. In order to involve the manager in the RTW process, the rehabilitation coordinator may use the dialogue-based workplace intervention. When the reason for sick leave is not work-related, consideration should be given to involving other actors such as social services or relatives.
